# Unravelling the origin of the common wall lizards (*Podarcismuralis*) in south-eastern Europe using mitochondrial evidence

**DOI:** 10.3897/BDJ.10.e90337

**Published:** 2022-09-30

**Authors:** Oleksandra Oskyrko, Tibor Sos, Emiliya Vacheva, Sabina E. Vlad, Dan Cogălniceanu, Tobias Uller, Nathalie Feiner, Miguel A. Carretero

**Affiliations:** 1 CIBIO Research Centre in Biodiversity and Genetic Resources, InBIO, Universidade do Porto, Campus de Vairão, 4485-661, Vairão, Portugal CIBIO Research Centre in Biodiversity and Genetic Resources, InBIO, Universidade do Porto, Campus de Vairão, 4485-661 Vairão Portugal; 2 Department of Zoology, Faculty of Science, Charles University, Vinićná 7, 12844, Prague, Czech Republic Department of Zoology, Faculty of Science, Charles University, Vinićná 7, 12844 Prague Czech Republic; 3 Evolutionary Ecology Group, Hungarian Department of Biology and Ecology, Babeș-Bolyai University, Clinicilor Street 5–7, 400006, Cluj Napoca, Romania Evolutionary Ecology Group, Hungarian Department of Biology and Ecology, Babeș-Bolyai University, Clinicilor Street 5–7, 400006 Cluj Napoca Romania; 4 “Milvus Group” Bird and Nature Protection Association, 540445, Tîrgu Mureș, Romania “Milvus Group” Bird and Nature Protection Association, 540445 Tîrgu Mureș Romania; 5 Institute of Biodiversity and Ecosystem Research, Bulgarian Academy of Sciences, 1 Tsar Osvoboditel Blvd, 1000, Sofia, Bulgaria Institute of Biodiversity and Ecosystem Research, Bulgarian Academy of Sciences, 1 Tsar Osvoboditel Blvd, 1000 Sofia Bulgaria; 6 Faculty of Natural and Agricultural Sciences, Ovidius University Constanţa, Aleea Universități 1, Campus - Corp B, 900470, Constanƫa, Romania Faculty of Natural and Agricultural Sciences, Ovidius University Constanţa, Aleea Universități 1, Campus - Corp B, 900470 Constanƫa Romania; 7 CEDMOG Center, Ovidius University Constanța, Tomis Avenue 145, Constanƫa, Romania CEDMOG Center, Ovidius University Constanța, Tomis Avenue 145 Constanƫa Romania; 8 Asociația Chelonia România, 062082, Bucharest, Romania Asociația Chelonia România, 062082 Bucharest Romania; 9 Department of Biology, Lund University, Sölvegatan 37, 223 62, Lund, Sweden Department of Biology, Lund University, Sölvegatan 37, 223 62 Lund Sweden; 10 Departamento de Biologia, Faculdade de Ciências da Universidade do Porto, R. Campo Alegre, s/n, 4169 - 007, Porto, Portugal Departamento de Biologia, Faculdade de Ciências da Universidade do Porto, R. Campo Alegre, s/n, 4169 - 007 Porto Portugal; 11 BIOPOLIS Program in Genomics, Biodiversity and Land Planning, CIBIO, Campus de Vairão, 4485-661, Vairão, Portugal BIOPOLIS Program in Genomics, Biodiversity and Land Planning, CIBIO, Campus de Vairão, 4485-661 Vairão Portugal

**Keywords:** phylogeography, genetic diversity, introduction, Eastern Europe, Lacertidae

## Abstract

The origin of the common wall lizards (*Podarcismuralis*) populations in south-eastern Europe (namely in Bulgaria and Romania), representing the north-eastern range border of this species, was addressed using mitochondrial DNA. We compared *cytochrome b* sequences from Bulgaria and Romania with those from the contiguous range in Central Europe that are available from previous studies. We recorded five main haplogroups in Bulgaria and Romania, belonging to the Central Balkan clade. However, haplogroup III was recorded in more localities than previously found. Additionally, signs of haplotype admixture were identified in several populations along the Danube River. The presence of the Southern Alps haplotype in one population from Otopeni, Bucharest (Romania) and its close phylogenetic relationships to north Italy populations suggests human-mediated introductions of this wall lizard clade in Romania. Our results confirm that *P.muralis* can have non-native lineages and admixture through active human-mediated transport.

## Introduction

Detailed phylogeographic data from widespread Western Palearctic species are particularly valuable for evaluating the plausibility of a scenario of Ice-age survival in refugia. In the recent geological past (Pleistocene), climate fluctuations have resulted in range shifts, leading to geographic isolation, genetic divergence and formation of more or less distinct lineages within well-defined species (Avise 1994, Davies and Shaw 2001, Hewitt 2004, Sommer and Zachos 2009). Reptiles are an interesting group to study with respect to biogeography and the evolution of local adaptation, particularly at the northern limit of the present-day ranges. In recent decades, phylogeographic studies have been used to assess the genetic consequences of Pleistocene Ice Ages on various organisms, highlighting the dynamic nature of species ranges and the role of micro-evolutionary processes in determining the extent and structure of intraspecific diversity (Hewitt 2000, Widmer and Lexer 2001, Schmitt 2007). Additionally, inferences from geographical organisation of genetic markers substantially contribute to historical and ecological biogeography, including identification of recent, human-mediated admixture (Lenk et al. 1999).

The genus *Podarcis* (Squamata, Lacertidae) comprises approximately 25 species (Speybroeck et al. 2020, Uetz et al. 2022). The origin of this taxon is hypothesised to have occurred in the Oligocene, while the diversification amongst the main lineages within this genus probably occurred during the Miocene (Yang et al. 2021). The *Podarcis* species with the largest range is the Common Wall lizard *Podarcismuralis* (Laurenti, 1768) (Arnold et al. 2007, Sillero et al. 2014). This species is distributed from the Iberian Peninsula to Asia Minor, but it is also native to extra-Mediterranean regions in estern, Central and Eastern Europe (Sindaco and Jeremenko 2008, Schulte et al. 2012a). The distribution of *P.muralis* is unusual relative to that of its congeners and, together with other characteristics, makes this species a useful model for evaluating the relative contribution of southern versus extra-Mediterranean refugia in shaping the current distribution of species and their genetic diversity. Previous studies, based on mitochondrial DNA data, suggested that such widespread geographic distribution has been accompanied by regional differentiation into more than 20 genetic lineages and several of them separated by low genetic divergence (short internal branches; Salvi et al. 2013). Recent phylogenomic studies suggest a much more complex scenario with a Miocenic origin in Italy, expansion to Iberia and the Balkans, secondary contacts and Quaternary subdivision in lineages (Yang et al. 2022). Moreover, it is a highly successful introduced species in north-western Europe, including England, where around 150 non-native *P.muralis* populations have been identified (Schulte 2008, Michaelides et al. 2015).

Currently, the species attains the north-eastern limit of its native range in Romania, occurring primarily along the Carpathian Mountains and in several sites in the Danube River valley and in the Dobruja region (Schulte 2008; Cogălniceanu et al. 2013). However, previous phylogeographic studies had limited data for south-eastern Europe and the resolution is therefore poor (Salvi et al. 2013, Jablonski et al. 2019, Yang et al. 2022). Of particular interest is the region along the Danube on the Romanian-Bulgarian border, where human-mediated colonisation has already been identified in Ukraine (Oskyrko et al. 2020). That study cast doubts on the native status of some Romanian populations and about the phylogeography of wall lizards in this area. Indeed, the current distribution pattern suggests either recent expansion or range collapse and it may even be possible that populations along the Danube are the results of recent colonisation, perhaps with the help of humans.

The aim of our study was, therefore, to ascertain the origin and population structure of *P.muralis* in south-eastern Europe and identify the biogeographic processes shaping the genetic diversity of lizards at its north-eastern range margin. We sampled lizards from 28 locations in Romania and Bulgaria to: (1) identify the geographic distribution of mitochondrial haplotypes and (2) determine whether or not there is evidence for recent introductions of *P.muralis*.

## Material and methods

**Figure 1.**
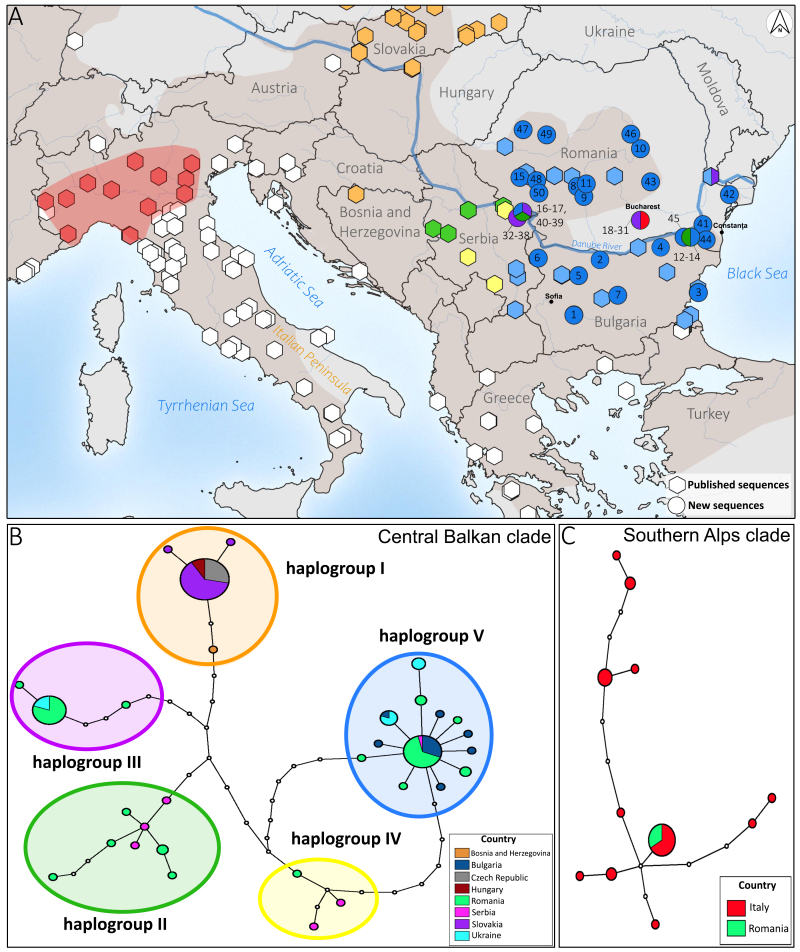
Map and networks of the natural distribution and introduced populations of *Podarcismuralis* in this study. **A** Geographical position of the main *cytb* haplogroups of the Central Balkan clade and Southern Alps clade in the studied area. Approximate species distribution is given in brown shading (Sillero et al. 2014). Colours of haplotypes follow colours from Jablonski et al. (2019): I haplogroup - orange, II haplogroup - green, III haplogroup - violet, IV haplogroup - yellow, V haplogroup - blue, Southern Alps clade - red. The numbers used for the samples in this study are listed in Table 1; **B.** The main haplogroups of the Central Balkan clade (Schulte 2008, Jablonski et al. 2019, this study). Colours correspond to the country of the specimen’s geographical origin and each circle corresponds to a haplotype. Each circle size is proportional to their frequencies and open circles represent missing haplotypes. The different colours within the network depict the principal identified haplotypes. Colours and numbering of haplotypes according to Jablonski et al. (2019); **C** The Southern Alps haplotypes network, designed from the *cytb* from 29 individuals of *P.muralis* (Giovannotti et al. 2010, Salvi et al. 2013, Michaelides et al. 2015, Jablonski et al. 2019, this study). Colours correspond to the country of the specimen’s geographical origin. Circle size is proportional to the number of samples under the same haplotype. Open circles represent missing haplotypes.

We sequenced a region of the *cytochrome b* (*cytb*) gene in the mitochondrial genome of 50 *P.muralis* individuals from 28 locations in Bulgaria and Romania. Seven samples were collected in Bulgaria (7 locations) and the remaining 43 in Romania (21 locations). Lizards were captured and the outer tip (~ 1 cm) of the tails was removed by gently squeezing with a pair of tweezers and stored in 96% ethanol (at a temperature of -80^0^C). All lizards were released at the capture location. The samples were collected during 2017-2021. The geographical coordinates were recorded with a hand-held GPS (Garmin Montana 700i and Garmin GPSMAP 64s). The geographic references are given in Table 1 and shown in Fig. 1.

DNA was extracted and approximately a 700 base pair (bp) region of the *cytb* gene was amplified following the same protocol as in previous works (Michaelides et al. 2013, Michaelides et al. 2015, Uller et al. 2019). Partial mitochondrial DNA (mtDNA) *cytb* gene was amplified by PCR using the primer pair LGlulk (5′-AACCGCCTGTTGTCTTCAACTA-3′) and Hpod (5′-GGTGGAATGGGATTTTGTCTG-3′) (Podnar et al. 2007, Schulte et al. 2012a, Michaelides et al. 2013) and the primers GluDG-L (5'- TGACTTGAARAACCAYCGTTG-3') and CB3H (5'-GGCAAATAGGAARTATCATTC-3') from Palumbi et al. (1991). Amplifications were carried out in a total volume of 15 μl consisting of 7.5 μl of MyTaq HS Mix, 0.45 μl (8 pmol) of each primer, 4.6 μl PCR-grade H_2_O and 2 μl template DNA. The PCR conditions were as follows: an initial denaturation step at 94°C for 1 min, followed by 34-35 cycles at 94°C for 1 min (or 30 s for some samples) , 53°C for 45 s or 52°C for 60 s and 72°C for 1 min and a final extension step at 72°C for 10 min (Jablonski et al. 2019 and Uller et al. 2019). Products were visualised with 1.5% agarose gel electrophoresis. The PCR products were purified using the ExoSAP-IT Cleanup Reagent (Applied Biosystems). The suitable amplicons were sent to external service (Beckman Coulter Genomics, Porto, Portugal) or processed in-house at Lund University (Lund, Sweden) for purification and Sanger-sequencing. New sequences used in this study were submitted to GenBank under the accession numbers ON666630-ON666679.

The sequences were corrected, aligned and trimmed to a uniform length of 656 bp in Geneious Prime v.2020.1 (https://www.geneious.com). The alignment was performed with MAFFT v.6 (Katoh et al. 2017). For the species-wide tree, we used 289 published sequences from all over the natural range (Poulakakis et al. 2005, Podnar et al. 2007, Schulte 2008, Giovannotti et al. 2010, Salvi et al. 2013, Michaelides et al. 2015, Jablonski et al. 2019, Oskyrko et al. 2020). For the Central Balkan clade, we used 84 sequences (MG851915-MG851983 and MN866797-MN866817) (Jablonski et al. 2019, Oskyrko et al. 2020). The 136 samples from the Italian Peninsula were used from Giovannotti et al. (2010), Salvi et al. (2013), Michaelides et al. (2015) and Jablonski et al. (2019) (FJ867365-FJ867394, KF372244-KF372225, KP972470-KP972539 and MG851980-MG851983). One sequence from *Podarcisliolepis* from GenBank was used as an outgroup (accession number KF372218), following Salvi et al. (2013). The best-fitting model was Hasegawa–Kishono–Yano+G (HKY+G) using Partition Finder 2 v.2.1 (Lanfear et al. 2017). Maximum Likelihood (ML) trees were constructed using IQ-TREE (Trifinopoulos et al. 2016) with 1000 pseudoreplicates to assess the confidence of branches. Bayesian Inference (BI) analysis was carried out by MrBayes v.3.2 (Huelsenbeck and Ronquist 2001) with 5×10^7^ generations and four chains and subsampling parameters and trees every 100 generations. Finally, 10% of the posterior samples were discarded as burn-in. The resulting trees were annotated using FigTree 1.4.3 (Rambaut 2014). To inspect the mtDNA *cytb* haplotype diversity, a 95% maximum parsimony haplotype network was constructed using the TCS inference in the programme TCS1.21 and tcsBU (Clement et al. 2000; Santos et al. 2016). Permutation tests (*p*-distances) were evaluated in R 4.2.0 (R Core Team 2020) using the “pegas” package (Paradis 2010). The map was created in QGIS 3.10.8 (Team 2020).

## Results

We obtained 50 complete *cytb* sequences with no signal of contamination or sequences of nuclear genomic origin. GenBank accession numbers for the sequences generated in this study are reported in Table 1. The total tree, which included 346 specimens from the *P.muralis* natural range, showed that most of our samples collected in Bulgaria and Romania were included in the Central Balkan clade (CB; Fig. 1, A). The average uncorrected genetic distance between mitochondrial clades was 4.2%. The Bayesian Inference (BI)/Maximum Likelihood (ML) analyses resulted in a phylogenetic tree for the CB clade (126 samples) with many distinct haplogroups (Suppl. material 1), which is in general concordance with previous studies (see discussion). The phylogenetic relationships inferred from the trees, based on the combined mitochondrial sequences, showed a well-supported clade (BP ≥ 90) with a geographic coherence. We received five well-supported haplogroups: I, II, III, IV and V from eight countries (Bosnia and Herzegovina, Bulgaria, Czech Republic, Hungary, Romania, Serbia, Slovakia and Ukraine). We did not find any new clades or haplogroups. Out of the 50 new sequences from Bulgaria and Romania, 45 fell into three of the distinct CB clades, II, III and V, previously known from Bulgaria, Serbia, Romania and Ukraine (Fig. 1). The phylogenetic networks depicting the relationships between haplotypes are shown in Fig. 1. These haplogroups are separated from each other by 0.2–1.2% of uncorrected p-distance in their *cytb* sequences. Most of our samples (n = 24) are included in the most common haplogroup V, which includes 13 haplotypes. This haplogroup is mainly composed of samples from northern and central Romania (17 localities), as well as all samples from Bulgaria (seven localities). However, nine samples from Lacu Morii, Bucharest (Romania) were included in haplogroup III. Additionally, the samples in the area of the Romanian villages of Svinita (n = 7) and Dubova (n = 1) were from this haplogroup as in previous studies. Only four of our specimens belonged to haplogroup II (three localities, eight haplotypes) and were collected from localities along the Danube. None of our new samples grouped with haplogroups I and IV.

For the first time, we collected five samples of *P.muralis* in Otopeni, Bucharest (Romania) and these samples were not included in the CB clade during analysis. The BI/ML analyses of these sequences revealed close affiliation with the Southern Alps clade, which has its main distribution in northern Italy (Suppl. material 2 and Fig. 1). Our samples closely grouped with haplotypes from north-eastern Italy (FJ867367 and KF372225-KF372229). Additionally, for the first time for Romania, we found three admixed populations: in Bucharest (two different haplogroups: III and Southern Alps (SA)), in Băneasa (two haplotypes: II and V) and the range of Valea Mraconia and Dubova (three different haplogroups: II, III and V that are included in CB clade).

## Discussion

The common wall lizard *Podarcismuralis* exhibits a complex phylogeographic pattern with multiple divergent mtDNA clades across its range. An early (Miocenic) diversification appears to have occurred in the south-central part of its current range, in what today is the Italian Peninsula, followed by an expansion out of Italy and subsequent lineage subdivision in the Iberian Peninsula, Central Europe and the Balkans (Salvi et al. 2013, Yang et al. 2022). However, it has been unclear to what extent the Central Balkan (CB) clade exhibits a well-defined geographic structure since it was sampled less thoroughly in previous studies (Schulte 2008, Schulte et al. 2012b, Salvi et al. 2013, Jablonski et al. 2019, Yang et al. 2022). In general, previous research suggested that the populations in Central Europe originated from the CB clade and not from the southern Carpathian refugia, as would be more likely given the phylogeographic patterns of several other reptile species (Salvi et al. 2013, Jablonski et al. 2019). However, due to a sampling gap in south-eastern Europe, these conclusions remain preliminary. Moreover, recent analyses of the genome-wide data have demonstrated extensive gene flow even between distantly-related mtDNA lineages of *P.muralis* (Yang et al. 2018, Yang et al. 2020, Yang et al. 2022).

Our results have added more clarity to the diversity of haplotypes in this region. We showed that the haplotype diversity was more often south of the Danube River, while the populations on the Bulgarian and northern edge of the Romania distributional range are relatively uniform (Fig. 1). A refugial area in the south Carpathians has already been suggested for many species showing high genetic diversity and distinct lineages in this area (Willis and Van Andel 2004, Kotlík et al. 2006, Ursenbacher et al. 2008, Hammouti et al. 2010). However, our results did not reveal isolated lines, but almost null or very shallow divergence amongst *P.muralis* populations within the mtDNA clades in this area. Yet, the use of nuclear markers (Psonis et al. 2018, Yang et al. 2020, Yang et al. 2022, Ruiz-Miñano et al. 2022) is needed for assessing more detailed patterns of variation.

**Figure 2.**
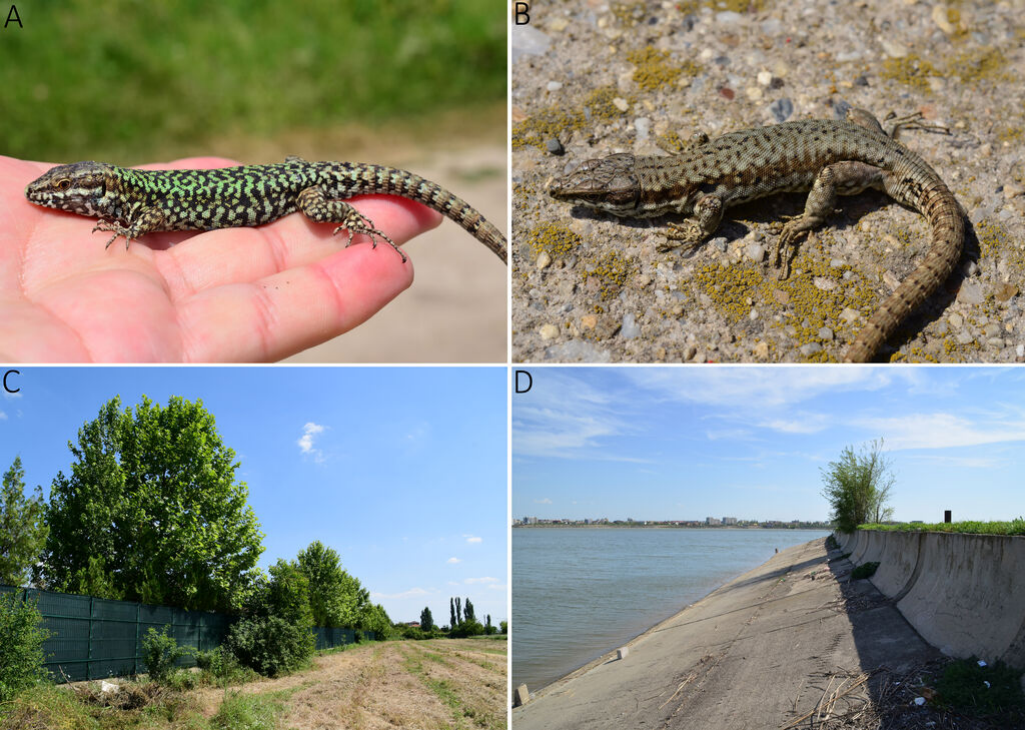
Representative pictures of *Podarcismuralis* and their habitats in Romania. **A** *P.muralis* from Otopeni, Bucharest (Romania); **B** *P.muralis* from Lacul Morii, Bucharest (Romania); **C** Otopeni, Bucharest (Romania); **D** Lacul Morii, Bucharest (Romania). Photos by T. Sos.

Our results suggest that a more precise understanding of the current distribution and demography of the isolated populations, in particular along the Danube River itself, (Romanian - Bulgarian border) that can be necessary to determine their history. Here, we identify a non-native population of *P.muralis* in Otopeni, Bucharest (Fig. 2, A and C), which were found to exhibit mitochondrial genotypes from the Southern Alps (SA) clade, a lineage whose native distribution is restricted to north-eastern Italy (Schulte 2008, Michaelides et al. 2015). This population is remarkable because it exhibits a bright green colouration in the males (Fig. 2). We have not found similar colouration for this species elsewhere in Romania and the colouration is compatible with the description of the subspecies *P.m.maculiventris* from Italy. However, *P.muralis* from the main mtDNA lineages are reproductively compatible (e.g. While et al. 2015, MacGregor et al. 2017, Yang et al. 2020) and introduced populations north of the native range margin are often admixed (Michaelides et al. 2015, Beninde et al. 2018, Michaelides et al. 2015). Thus, it is possible that this population represents admixture between CE and SA lineages.

Most likely, the SA origin of the Otopeni population is a result of human-mediated transport on inland waterway vessels with construction materials, plants or other goods, as was discovered in other countries (Hedeen and Hedeen 1999, Gherghel et al. 2009, Santos et al. 2019). This introduction resembles another record of *P.muralis* in the southern part of Ukraine (Reni City) that were introduced from different source populations, but both occurring within the "Central Balkan clade" (Oskyrko et al. 2020). We also found a CB (haplogroup III) in another population from Lacul Morii, Bucharest (Fig. 2, B and D), which likely is another recent introduction. Multiple native-range sources are a common characteristic of biological invasions (Dlugosch and Parker 2008), including for invasive lizards (Kolbe et al. 2004, Kolbe et al. 2007, Chapple et al. 2012, Schulte et al. 2012a). Other cryptic introductions of different lineages are likely within the native range of the species, but this can be difficult to identify without large sample sizes.

In summary, our results suggest a rather homogeneous genetic structure within the easternmost part of the distribution of the *P.muralis*. Recent human introductions are, however, expanding the species range and resulting in introductions of different lineages, showing the importance of documenting cryptic introductions and investigating their sources and pathways to avoid further possible invasions.

## Supplementary Material

9E912561-1EA5-5632-8657-7D6230B8F2D1 10.3897/BDJ.10.e90337.suppl1 Supplementary material 1 Bayesian Inference consensus tree derived from mitochondrial *cytb* sequences showing schematically overall genetic diversity of the species and details of the Central Balkan clade
Data type Phylogenetic Brief description Bootstrap values (> 50%) are indicated above nodes of major clades. Numbers at nodes show Bayesian posterior probabilities. Colours of haplotypes follow colours from Jablonski et al. (2019). New samples are highlighted in red. File: oo_714253.pdf https://binary.pensoft.net/file/714253 Oleksandra Oskyrko, Tibor Sos, Emiliya Vacheva, Sabina E. Vlad, Dan Cogălniceanu, Tobias Uller, Nathalie Feiner, Miguel A. Carretero

30B2ECFE-DA9A-5267-8619-262CAFB399CD 10.3897/BDJ.10.e90337.suppl2 Supplementary material 2 Bayesian Inference consensus tree derived from mitochondrial *cytb* sequences showing schematically overall genetic diversity of the species and details of the Southern Alps clade Data type Phylogenetic Brief description Bootstrap values (> 50%) are indicated above nodes of major clades. Numbers at nodes show Bayesian posterior probabilities. New samples are highlighted in red. File: oo_714254.pdf https://binary.pensoft.net/file/714254 Oleksandra Oskyrko, Tibor Sos, Emiliya Vacheva, Sabina E. Vlad, Dan Cogălniceanu, Tobias Uller, Nathalie Feiner, Miguel A. Carretero

## Figures and Tables

**Table 1.** Tissue samples and sequences of *Podarcismuralis* used in this study.

№	**GenBank accession** **number**	**Country**	**Locality**	**Coordinates**		**Haplogroup**	**Year of collection**
				**N**	**E**		
1	ON666630	Bulgaria	Gabrovitsa	42.263	23.920	V	2018
2	ON666631	Bulgaria	Pleven	43.423	24.611	V	2017
3	ON666632	Bulgaria	Obzor	42.789	27.885	V	2020
4	ON666633	Bulgaria	Isperikhovo	43.714	26.921	V	2020
5	ON666634	Bulgaria	Karlukovo	43.180	24.068	V	2021
6	ON666635	Bulgaria	Falkovets	43.586	22.783	V	2021
7	ON666636	Bulgaria	Shejnovo	42.685	25.308	V	2020
8	ON666637	Romania	Bistriţa Vâlcea	45.213	24.030	V	2021
9	ON666638	Romania	Capu Dealului	44.990	24.237	V	2021
10	ON666639	Romania	Turia	46.055	26.042	V	2021
11	ON666640	Romania	Căciulata Vâlcea	45.272	24.315	V	2021
12	ON666641	Romania	Băneasa	44.068	27.646	II	2021
13	ON666642	Romania	Băneasa	44.068	27.646	II	2021
14	ON666643	Romania	Băneasa	44.068	27.646	V	2021
15	ON666644	Romania	Caransebeş	45.417	22.196	V	2020
16	ON666645	Romania	Valea Mraconia	44.639	22.283	V	2020
17	ON666646	Romania	Valea Mraconia	44.639	22.283	II	2020
18	ON666647	Romania	Otopeni, Bucharest	44.563	26.063	Southern Alps	2021
19	ON666648	Romania	Otopeni, Bucharest	44.563	26.063	Southern Alps	2021
20	ON666649	Romania	Otopeni, Bucharest	44.563	26.063	Southern Alps	2021
21	ON666650	Romania	Otopeni, Bucharest	44.563	26.063	Southern Alps	2021
22	ON666651	Romania	Otopeni, Bucharest	44.563	26.063	Southern Alps	2021
23	ON666652	Romania	Lacul Morii, Bucharest	44.453	26.038	III	2019
24	ON666653	Romania	Lacul Morii, Bucharest	44.453	26.038	III	2019
25	ON666654	Romania	Lacul Morii, Bucharest	44.453	26.038	III	2019
26	ON666655	Romania	Lacul Morii, Bucharest	44.453	26.038	III	2019
27	ON666656	Romania	Lacul Morii, Bucharest	44.453	26.038	III	2019
28	ON666657	Romania	Lacul Morii, Bucharest	44.453	26.038	III	2019
29	ON666658	Romania	Lacul Morii, Bucharest	44.456	26.036	III	2019
30	ON666659	Romania	Lacul Morii, Bucharest	44.456	26.036	III	2019
31	ON666660	Romania	Lacul Morii, Bucharest	44.456	26.036	III	2019
32	ON666661	Romania	Şviniţa	44.501	22.104	III	2019
33	ON666662	Romania	Şviniţa	44.501	22.104	III	2019
34	ON666663	Romania	Şviniţa	44.501	22.104	III	2019
35	ON666664	Romania	Şviniţa	44.501	22.104	III	2019
36	ON666665	Romania	Şviniţa	44.501	22.104	III	2019
37	ON666666	Romania	Şviniţa	44.501	22.104	III	2019
38	ON666667	Romania	Şviniţa	44.499	22.101	III	2019
39	ON666668	Romania	Dubova	44.519	22.193	II	2019
40	ON666669	Romania	Dubova	44.634	22.279	III	2019
41	ON666670	Romania	Cernavodă	44.353	28.037	V	2018
42	ON666671	Romania	Agighiol	45.032	28.880	V	2018
43	ON666672	Romania	Pătârlagele	45.318	26.366	V	2018
44	ON666673	Romania	Negrești	44.004	28.140	V	2018
45	ON666674	Romania	Bugeac Lake	44.067	27.434	V	2018
46	ON666675	Romania	Ciba	46.377	25.735	V	2020
47	ON666676	Romania	Moneasa	46.473	22.299	V	2018
48	ON666677	Romania	Gura Zlata	45.344	22.731	V	2019
49	ON666678	Romania	Câmpeni	46.360	23.050	V	2019
50	ON666679	Romania	Cloșani	45.067	22.803	V	2020
