# Supplementary material for: Unravelling the origin of the common wall lizards (Podarcismuralis) in south-eastern Europe using mitochondrial evidence
Source: Biodivers Data J. 2022 Sep 30;10:e90337. doi: 10.3897/BDJ.10.e90337 (PMC9836615; doi:10.3897/BDJ.10.e90337)
Supplement: Supplementary material 1 — Bayesian Inference consensus tree derived from mitochondrial cytb sequences showing schematically overall genetic diversity of the species and details of the Central Balkan clade [file bdj-10-e90337-s001.pdf]

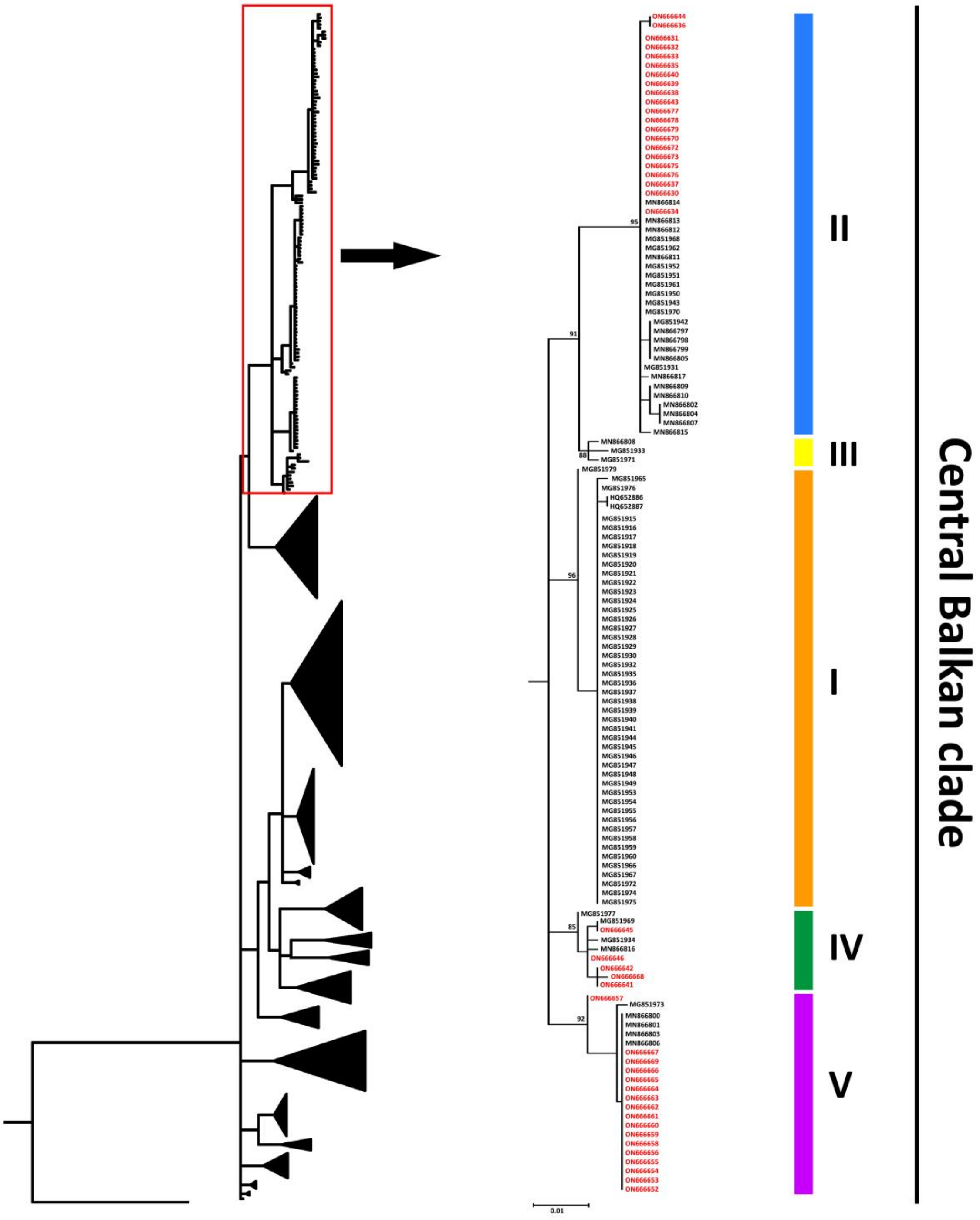

Figure S1. Bayesian Inference consensus tree derived from mitochondrial *cytb* sequences showing schematically overall genetic diversity of the species and details of the Central Balkan clade. Bootstrap values (>50%) are indicated above nodes of major clades. Numbers at nodes show Bayesian posterior probabilities. Colors of haplotypes follow colors from Jablonski et al. (2019). New samples are highlighted in red.
